# The Plaque Microbiota Community of Giant Panda (*Ailuropoda melanoleuca*) Cubs With Dental Caries

**DOI:** 10.3389/fcimb.2022.866410

**Published:** 2022-04-28

**Authors:** Rui Ma, Rong Hou, Jun-Liang Guo, Xiu-Yue Zhang, San-Jie Cao, Xiao-Bo Huang, Rui Wu, Yi-Ping Wen, Qin Zhao, Sen-Yan Du, Ju-Chun Lin, Yu Bai, Qi-Gui Yan, Dun-Wu Qi

**Affiliations:** ^1^College of Veterinary Medicine, Sichuan Agricultural University, Chengdu, China; ^2^Sichuan Key Laboratory of Conservation Biology for Endangered Wildlife, Chengdu Research Base of Giant Panda Breeding, Chengdu, China; ^3^Key Laboratory of Animal Disease and Human Health of Sichuan Province, Sichuan Agricultural University, Chengdu, China; ^4^Key Laboratory of Bio-Resources and Eco-Environment, Ministry of Education, College of Life Science, Sichuan University, Chengdu, China

**Keywords:** giant panda, caries, dental plaque, captive wildlife, microflora

## Abstract

Dental caries severely hinders efficient access to adequate energy in wildlife. Different food supplies will develop characteristic plaque, and the microorganisms of these plaque are closely related to dental health. Here, plaque samples from panda cubs with caries and caries-free were collected for 16S rRNA high-throughput sequencing. All sequences clustered into 337 operational taxonomic units (OTUs; 97% identity), representing 268 independent species belonging to 189 genera, 98 families, 51 orders, 24 classes, and 13 phyla. Two groups shared 218 OTUs, indicating the presence of a core plaque microbiome. α diversity analysis showed that the microbial diversity in plaques with caries exceeded that of caries-free. The dominant phyla of plaque microbiota included Proteobacteria, Bacteroidetes, Firmicutes, Fusobacteria, and Actinobacteria. The dominant genera included *unclassified Neisseriaceae*, *Actinobacillus*, *Lautropia*, *Neisseria*, *Porhyromonas*, *unclassified Pasteurellaceae*, *Moraxella*, *Streptococcus*, *Bergeywlla* and *Capnocytophaga*. β diversity analysis showed that the plaque microbial community structure was different between two groups. Using LEfSe analysis, 19 differentially abundant taxa were identified as potential biomarkers. Finally, function predictions analysis showed All the energy related metabolic pathways on KEGG level 2 were enriched in caries-active group. Consistent with the mainstream caries-causing narrative, our results illuminate the lack of information regarding the oral microflora composition and function within giant panda cubs.

## Introduction

Dental caries, as irreversible tooth decay, may cause pulpitis, severe toothache, chronic infections and eventually lead to health problems such as anorexia, difficulty foraging and malnutrition, as well as gastrointestinal, cardiovascular, renal and other systemic disorders (Selwitz et al., 2007; Maruyama et al., 2014). After years of research, the etiology and related risk factors of human dental caries have been studied in detail and explained from the perspective of oral microbes (Fleming, 1965; Tanzer et al., 2001; Munson et al., 2004). Nevertheless, only a few studies have focused on dental disease in animals. Except those which have focused on pathogenic animal models (Loyola and Leyva, 1990), a small group have focused on dental caries of companion and domestic animals (Sturgeon et al., 2012; Sturgeon et al., 2014). There have been very few studies of caries in wild animal species (Cuozzo and Sauther, 2006; Li et al., 2013; Malmsten et al., 2015), especially in giant pandas (Wang, 1961; Jin et al., 2012).

With a unique digestive system and foraging patterns, giant pandas spend more than 10 hours per day eating hard bamboo, as a result complete and healthy teeth are especially important for this rare species of bear (Hull et al., 2011). Dental caries is one of the most harmful diseases affecting the quality and health of both wild and captive pandas (Jin et al., 2012; Jin et al., 2015). The prevalence of dental caries of ancient pandas was about 25% (Wang, 1961). The rate of caries of wild pandas living in Qinling mountain, Shaanxi Province was 22% (Shuangyun et al., 2009) and captive born individuals at Beijing Zoo reached 62.5% (Jin et al., 2012). This rate is much higher than the prevalence of caries reported for black bears (*Ursus americanus*) (11%) living in Northern Wisconsin (Manville, 1978), wild brown bears (*Ursus arctos*) (11.2%) and Alaskan grizzlies bears (*Ursus arctos horribilis*) (14.7%) living in Switzerland (Wenker et al., 1999). Dental caries has become an urgent problem for captive pandas, especially amongst cubs.

Breast milk is the only food source for wild giant pandas until they are weaned at 1.5 years of age, after which their diet will consist of more than 99% bamboo (Guo et al., 2018). On the contrary, formula milk will be suppled to ensure captive giant panda cubs have enough nutrition for growth before being weaned. Artificial formula is very different from breast milk in terms of composition and nutrition, such as the species and percentage of carbohydrates (Xuanzhen et al., 2005). Due to the high correlation between the development of dental plaque and food composition, the various species and percentage of compounds in the food will create the specific dental plaque. Compared with wild individuals, captive giant pandas are more likely to have caries due to higher proportions of concentrate as part of their daily food intake and fruit, which may increase the intake of carbohydrates (Stromquist et al., 2009; Jin et al., 2012). The diet of panda cubs is particularly monotonous, except for breast milk. According to the ecological plaque hypothesis, dental plaque biofilm becomes pathogenic when external challenges drive it towards a state with a high proportion of acid-producing bacteria (Xie et al., 2010; Head et al., 2014). With the rapid development of biotechnology and bioinformatics methods in recent years, high-throughput sequencing has been proven useful for a better understanding of both cultured and un-cultured bacteria and the microbiota-related diseases, which has opened the way toward a new understanding of the composition and structure of an animal’s microbiomes and the factors that affect them (Teng et al., 2015).

Currently, there is no study investigating the difference of the oral bacterial community between giant panda cubs with healthy teeth and those with caries. Therefore, based on 16S rRNA gene sequencing, the present research is aimed to increase the understanding of giant panda caries by comparing the dental plaque microbiota between healthy and caries-active giant panda cubs.

## Materials and Methods

### Animal Management and Welfare

All of the giant panda cubs in this study were socially housed in outdoor enclosures during the day and returned to an indoors den to stay with their mother at night. The procedures for sample collection and handling were approved by the Institutional Animal Care and Use Committee of the Chengdu Research Base of Giant Panda Breeding (IACUC No. 2018).

### Samples

In this study, eight captive panda cubs born in 2017 at the Chengdu Research Base of Giant Panda Breeding were selected. Based on the veterinarian’s clinical diagnosis of dental health, the eight cubs were divided into two groups when they reached 1 year old: Caries-active group (CA, n = 4) and Caries-free group (CF, n = 4). In order to ensure that the sequencing results can truly reflect the composition of dental plaque microbiota, sampling was performed 4 h after eating in the afternoon between 17:00 – 18:00. All individuals had no record of antibiotic use in nearly three months before sampling. All sample collections were performed without anesthetic or restraint benefiting from the trust of the cubs with their keepers. Briefly, sterile swabs were used to collect dental plaque sample from all the tooth surfaces from giant panda avoiding contamination from gingival bleeding. Each swab with a plaque sample was immediately released into a sterile, labeled EP tube, and then immediately placed in a transfer box with dry ice. All samples were transported on dry ice to our laboratory for DNA extraction.

### DNA Extraction and 16S rRNA Gene Sequencing

DNA was isolated from the sample swabs using the procedure described by Verdon et al. (Verdon et al., 2014) with the Tiangen TIANamp Swab DNA Kit (Tiangen, China) according to the manufacture’s instruction for isolation of DNA. The DNA quality of each sample was determined by 1% agarose gel electrophoresis, and sent to Shanghai Majorbio Bio-pharm Technology Co., Ltd (Shanghai, China) for further concentration assessment, amplified and sequenced according to their standard procedures (Wang et al., 2016; Yin et al., 2016). The V3–V4 regions of the 16S rRNA gene were amplified using the primers 338F (5’-ACTCCTACGGGAGGCAGCAG-3’) and 806R (5’-GGACTACGCGGGTATCTAAT-3’) that targeted conserved sequences found in bacteria. PCR reactions were performed in triplicate 20 μL mixture containing 4 μL of 5× FastPfu Bufer, 2μL of 2.5 mM dNTPs, 0.8 μL of each primer (5 μM), 0.4 μL of FastPfu Polymerase and 10 ng of template DNA. The amplicons were then extracted from 2% agarose gels and further purified by using the AxyPrep DNA Gel Extraction Kit (Axygen Biosciences, Union City, CA, USA) and quantified by QuantiFluor-ST (Promega, USA) according to the manufacture’s protocols. Purified amplicons were pooled in equimolar and paired-end sequenced (2 × 300) on an Illumina MiSeq platform (Illumina, San Diego, USA) according to the instruction. The raw reads were deposited into the NCBI Sequence Read Archive (SRA) database (Accession Number: SRP257211).

### Statistical Analysis

Raw reads were demultiplex and quality-filtered using QIIME (version 1.9.1). The taxonomy of each 16S rRNA gene sequence was analyzed against the SILVA 128/16s bacteria database with a confidence threshold of 70%. All operational taxonomic units (OTUs) at 97% identity were obtained using UPARSE (version 7.0). The Venn diagram and bar graph of each group was displayed by R software (v3.1.1). The Alpha diversity index was calculated by Mother (v.1.31.2), and A ranked abundance curve was drawn by R software (v3.1.1) to explain both the richness and evenness of species, and the Wilcoxon rank sum test were performed on Alpha diversity index between groups to obtain the difference of species diversity between two groups. Unweighted pair-group method with arithmetic means (UPGMA) cluster analysis with Bary-curtis distance matrix was performed by R software (V3.5.1). Beta diversity was compared with the representative sequences of OTUs for each group using the Bray-Curtis similarity coefficient by Vegan package of R (V3.5.1) and the difference between two groups was performed using Adonis. After 999 iterations, the Principal Co-ordinates Analysis (PCoA) display diagram were obtained, which were used to study the similarity or dissimilarity of sample community composition between samples. R (v3.5.1) was used to calculate the significance of the difference test between the two groups for the bacteria in the top 10 relative abundance. LEfSe software1 was used to make a LEfSe cluster diagram and an LEfSe linear discriminant analysis (LDA) diagram. Partial Least Squares Discriminant Analysis (PLS - DA) model analysis was performed by R software (V3.5.1). Receiver Operating Characteristic Curve (ROC) model analysis was performed by Graphpad Prism 9. A Wilcoxon test was used to look for differences between groups by R (v3.4.1). Microbial functions were predicted using PICRUSt (version 1.0.0) and aligned to the Kyoto Encyclopedia of Genes and Genomes (KEGG) database. In this study, differences were considered significant when *P < 0.05* and extremely significant when *P < 0.01*.

## Results

### Sequencing Date

**Figure 1:**
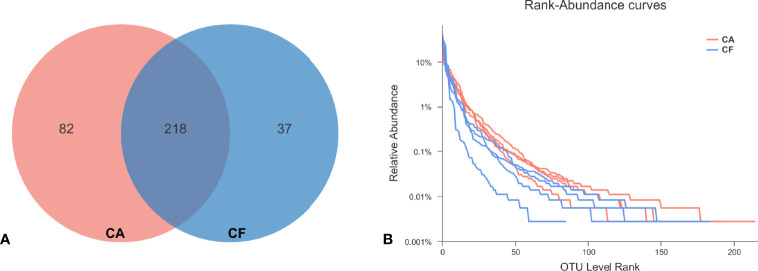
**(A)** OUT distribution in two groups. The red and blue circles represented samples of dental plaque in the CA and the CF group, respectively. The overlap denoted the OTUs shared by two groups. **(B)** Rank-Abundacnce curve. Curves of different colors show the relative percentage of the number of species (Y-axis) of each sample rank of the number of species (X-axis). The position of the horizontal coordinate at the end of the extension of the sample curve is the number of species in the sample. When the curve declines smoother indicates that the species diversity of the sample is higher, while the rapid and steep decline of the curve indicates that the proportion of dominant colonies in the sample is high and the diversity is lower.

After the raw data was filtered and spliced, a total of 350,420 high-quality tags were produced in the CA and CF groups, with an average of 43,802 sequences per sample (ranging from 41,246 to 46,372, **Table S1**). The average sequence length was 426 bp, with the maximum length being 486 bp and the shortest length being 209 bp. The total numbers of OTUs obtained was 337, among which 218 OTUs were shared by both groups, 82 and 37 OTUs were uniquely identified in the CA and the CF groups, respectively (**Figure 1A**).

### Microbial Diversity Analysis

The Chao1 and ACE richness index was higher in CA group than in CF group, but there was no significant difference between groups by Wilcoxon test (**Table 1**). However, the Shannon diversity index was significantly different between groups CA and CF (3.03 vs. 2.30, *p = 0.03*), the Simpson diversity index was significantly different between groups CA and CF (0.09 vs. 0.18, *p = 0.03*), demonstrating the higher bacterial diversity of dental plaques with caries compared to caries-free (**Table 1**). Good’s coverage estimator for each group was over 95%, indicating that the current sequencing depth was sufficient to saturate the bacterial diversity of dental plaques. In addition, Simpson index indicated that the bacterial-community distribution in the plaque samples was very uneven, which was also observed in the rank-abundance curve which had a high slope and a long tail comprised of relative low abundance OTUs (**Figure 1B**). The 100 most abundant OTUs represented 99.14% of all sequences. Most of the remaining OTUs were present at relative low abundance.

**Table 1:** Alpha diversity indices for dental plaque bacteria in each group at 97% identity.

Group	Chao1		Ace		Shannon		Simpson		Coverage	
	Mean	SD	Mean	SD	Mean	SD	Mean	SD	Mean	SD
CA	217.64	26.53	229.19	18.82	3.03^*^	0.17	0.09^*^	0.02	1.00	0.00
CF	171.33	35.30	169.06	41.50	2.30^*^	0.38	0.18^*^	0.05	1.00	0.00

SD, Standard Deviation. Asterisk indicates significant differences (p < 0.05, Wilcoxon). *Shannon index between CA and CF was statistically significant different (p = 0.03). *Simpson index between CA and CF was statistically significant different (p = 0.03).

### Community-Composition Analysis

**Figure 2:**
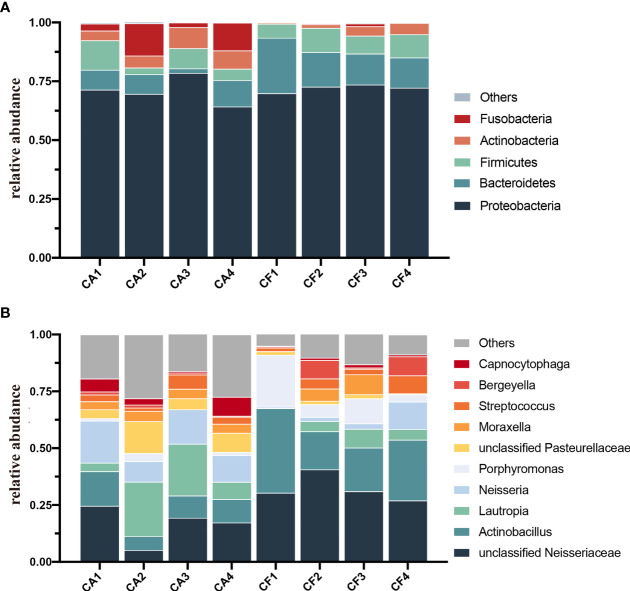
Microbial community bar plot. **(A)** Microbial community bar plot at the phylum level. Bar charts showing the relative abundance of all phyla detected in the dental plaque collected from the giant panda cubs in the CA (CA1-CA4) and the CF group (CF1-CF4). The identities of the microbiome were shown with color blocks on the right. **(B)** Microbial community bar plot at the genus level. Bar charts showing the relative abundance of all genera detected in the dental plaque collected from the giant panda cubs in the CA (CA1 – CA4) and the CF group (CF – CF4). The identities of the microbiome were shown with color blocks on the right.

**Figure 3:**
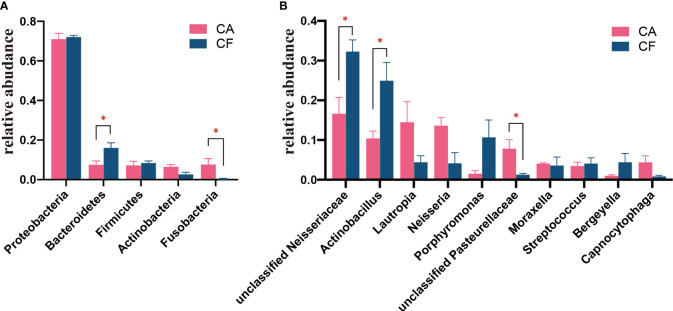
**(A)** Comparison of bacterial differences among diffierent groups of samples at the phylum level in top 5. **(B)** Comparative analysis of different species of bacteria among groups at genus level in top 10 (*0.01*<*p*<= *0.05*, marked with "*"; if *p*>*0.05*, not marked).

The bacterial distribution was characterized in terms of the relative taxonomic abundances. A total of 13 phyla, 24 classes, 51 orders, 98 families, 189 genera and 268 species were detected in the plaque samples. When comparing at the phylum level, the CA and CF groups shared the same top five bacterial species with little difference in overall relative abundance. The most dominant phylum of samples in the CA group was Proteobacteria (70.97%), followed by Bacteroidetes (7.49%), Firmicutes (7.18%), Fusobacteria (7.62%), and Actinobacteria (6.48%; **Figure 2A**). Proteobacteria (72.11%) was also the most abundant phylum in the CF samples, followed by Bacteroidetes (16.10%), and Firmicutes (8.41%), Actinobacteria (2.72%), Fusobacteria (0.41%; **Figure 2A**). The estimated cumulative relative abundance of these five dominant phyla was above 99% of the identified OTUs. The dental plaque bacterial community of giant panda cubs in the CA and CF groups were similar in composition. However, there was the discrepancy in their relative abundance at phyla level. Further analysis of the relative abundance showed that the relative abundance of Bacteroidetes in the CF group was significantly higher than that in the CA group (*p = 0.03*); whereas, the relative abundance of Fusobacteria in the CA group was significantly higher than that in the CF group (*p = 0.03*; **Figure 3A**).

At the genus level, a total of 189 bacterial genera were identified in our study, 171 in the CA group and 143 in the CF group. Dental plaque samples in CA group had the highest unclassified *Neisseriaceae* (16.61%) content, followed by *Lautropia* (14.48%), *Neisseria* (13.61%), *Actinobacillus* (10.38%) and unclassified *Pasteurellaceae* (7.81%). While dental plaque samples in the CF group displayed the highest amount of unclassified *Neisseriaceae*, accounting for 32.28% of the total microbial relative abundance, the second most abundant was *Actinobacillus* (24.95%), followed by *Porphyromonas* (10.67%), *Bergeyella* (4.41%) and *Lautropia* (4.40%; **Figure 2B**). However, the plaque bacterial communities in the CF group of giant panda cubs were notably different compared to the CA groups and had fewer number of species. In accordance with analysis of the relative abundance at genus level, the top 10 species were selected to evaluate the significance of the difference test between the two groups. The results showed that there were differences in unclassified *Neisseriaceae*, *Actinobacillus* and unclassified *Pasteurellaceae* between these two groups. The relative abundance of unclassified *Neisseriaceae* and *Actinobacillus* in the CF group was significantly higher than that in the CA group (*p = 0.03*); unclassified *Pasteurellaceae* in the CA group was significantly higher than that in the CF group (*p = 0.03*; **Figure 3B**). Our results showed that the dental plaque bacterial community in caries-active group is different with those in caries-free group at the genus level.

### Bacterial Community Structure

**Figure 4:**
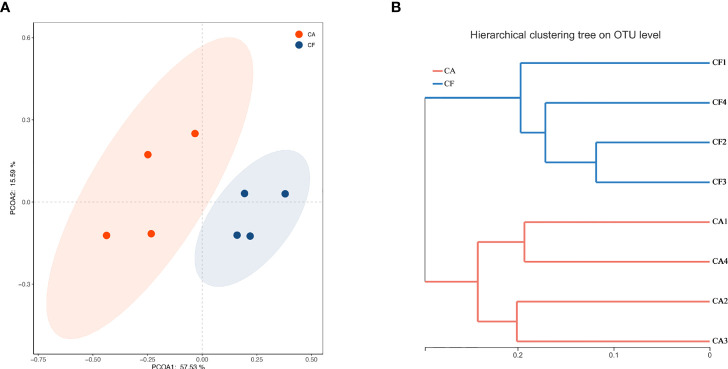
**(A)** PCoA based on the Bray-curtis distance matrix to find principal coordinates. (Description) X-axis, PCoA axis1 and Y axis, PCoA axis2. The scale of the X-axis and theY-axis are the projection coordinates of the sample points in the two-dimensional plane, respectively. A dot represents each sample, and different colors represent different groups. **(B)** Samples Clustering result (Description, Bray-curtis). The same color represents the samples in the same group. Short distance between samples represents high similarity.

To gain insight into similarities in the bacterial community structures between the two study groups, PCoA of beta diversity analysis was performed based on the Bray-curtis distances, which demonstrated different community structures among CA and CF groups. No sample’s microbiota overlapped with others and, a clear segregation in community structures was exhibited between the two groups, with the first two principal components representing 57.53% and 15.59% of the total variations (**Figure 4A**). Adonis testing confirmed that a significant separation occurred between the two groups (R^2^ = 0.484, *p = 0.027*). Unweighted pair-group method with arithmetic means (UPGMA) cluster analysis also revealed that the samples formed well-separated clusters corresponding to the two groups, indicating a difference in the bacterial community structures (**Figure 4B**).

### Core Microbiome

To examine the existence of an identifiable common core microbiome, we defined a core as the group of members shared among the microbial community and represented the core by overlapping areas in the circles in a Venn diagram, at 97% identity. We identified 218 OTUs which were shared between two groups, occupying 64.69% of all OTUs (337 OTUs) and 99.26% of all OUT relative abundance (**Figure 1A**). Furthermore, 11 shared phyla (84.62%), 20 shared classes (83.33%), 38 shared orders (74.51%), 72 shared families (73.47%), and 125 shared genera (66.14%). These shared taxonomic members can be regarded as the core microbiome of dental plaques.

**Figure 5:**
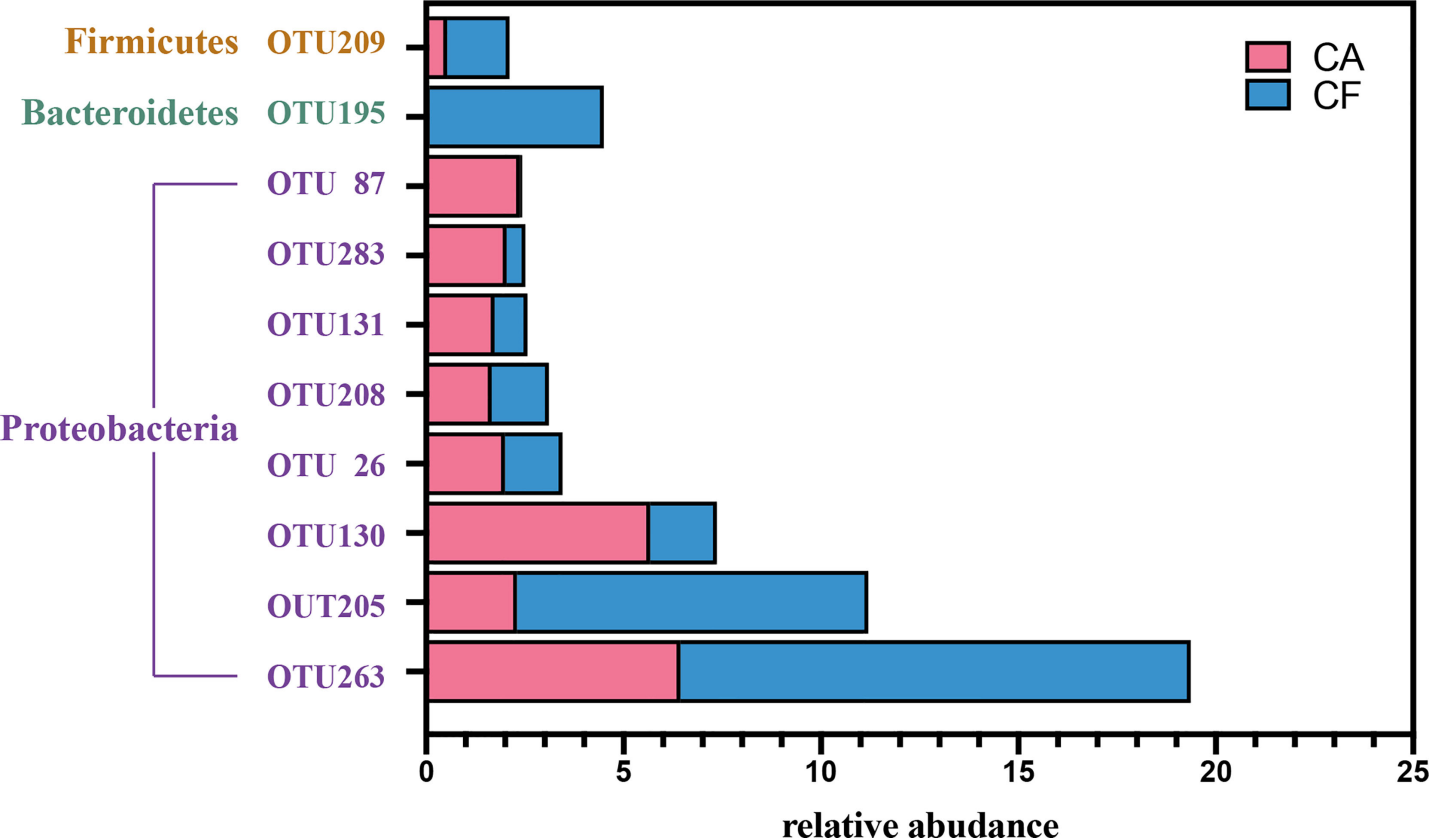
The barplot of top 10 relative abudance OTUs in core microbiota. Red color represent Caries-active group, blue color represent Caries-free group.

Among the 218 core OTUs, the 10 most relative abundant OTUs were *OTU 263* (19.36%), *OTU 205* (11.20%), *OTU 130* (7.36%), *OTU 195* (4.50%), *OTU 26* (3.45%), *OTU 208* (3.11%), *OTU 131* (2.56%), *OTU 283* (2.52%), *OTU 87* (2.43%) and *OTU 209* (2.12%) (**Figure 5**). The top 10 relative abundance OTUs in core microbiome accounted for 58.61% of all sequences. It is worth mentioning that among the top 10 relative abundance OTUs in core microbiome, except *OTU 209* belongs to Phylum Firmicutes and *OTU 195* belongs to Phylum Bacteroidetes, other 8 OTUs all belong to Phylum Proteobacteria. In the Venn diagram, the unique OTUs in each group were also observed with 82 and 37 unique OTUs found in the CA and CF groups, respectively. These unique OTUs were in relative low abundance, only containing 1–378 sequences, and can be considered as a variable microbiome.

### Potential biomarkers

**Figure 6:**
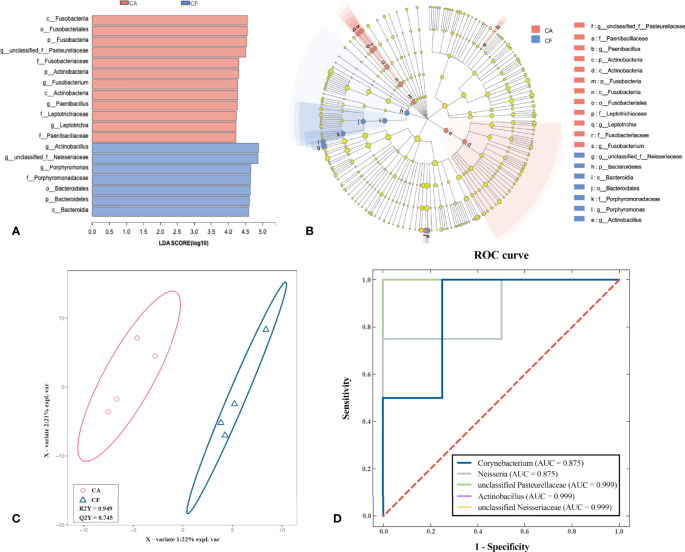
Identification of potential biomarkers using the LEfSe test, PLS-DA and ROC curve. **(A)** Linear discriminant analysis (LDA) demonstrated distinct microorganism enriched in the CA group and the CF group. The graph shows the LDA scores obtained from linear regression analysis of the significant microorganism groups in the two groups. When the default LDA value is more than 4.0 and the p value is less than 0.05, the result corresponds to a differential species. **(B)** Linear discriminant effect size cluster tree for 16S rRNA gene sequencing analysis. Different colors indicate different groups. Colored notes represent a group, color shading over the notes indicate the significant microbe biomarker in the group causing a significant difference in abundance, and the biomarker name is listed in the upper right corner. The yellow notes represent the biomarkers that do not show significant differences in abundance in the groups. (red indicating CA, blue indicating CF and yellow indicating non-significant). **(C)** Partial Least Squares Discrimination Analysis (PLS-DA) result. (Description) X-axis, X - Variate 1 and Y axis, X- Variate 2. The scale of the X-axis and theY-axis are the projection coordinates of the sample points in the two-dimensional plane, respectively. A dot represents each sample, red circle represents CA group and blue triangle represents CF group. **(D)** Receiver Operating Characteristic Curve (ROC) for screening for potential biomarkers. (Description) X-axis, 1 - Specificity and Y axis, Sensitivity. The red dotted line represents the random selection probability, Area Under Curve (AUC) represents the distinction of different categories.

To identify potential biomarkers, LEfSe analysis, PLS – DA analysis and ROC curve model was performed. The cladogram of the LEfSe analysis was showed in **Figure 6B**. The red and blue parts represented the groups of the CA and CF, respectively. The red and blue nodes in the cladogram denoted the bacteria playing a critical role in the CA and CF group, respectively. The yellow nodes corresponded to the bacteria that did not have an important role in each group. The LDA results showed that there was a significant difference between the genera in the dental plaque samples from the CA group and the CF group (**Figure 6A**). The genera found in the CA group belonged to unclassified *Pasteurellaceae*, *Fusobacterium*, *Paenibacillus* and *Leptotrichia* while the genera found in the CF group belonged to *Actinobacillus*, unclassified *Neisseriaceae* and *Porphyromonas*. Since LEfSe was a strict tool, we also generated a PLS-DA model to identify potential biomarkers on genus level more strictly. The key genera with VIP > 1 and *p < 0.05* were considered to be important contributors to the model. As shown in **Figure 6C**, a clear segregation in community structures was exhibited by PLS – DA model between the two groups. a total of 5 genera with a VIP score > 1 and *p < 0.05* were identified as key genera responsible for significant differences in the community composition. Among them, 3 genera were significantly enriched in CA group, including unclassified *Pasteurellaceae* (VIP = 3.644, *p = 0.03*), *Neisseria* (VIP = 4.815, *p = 0.03*) and *Corynebacterium* (VIP = 1.968, *p = 0.04*). The other 2 genera were significantly more abundant in CF group, including unclassified *Neisseriaceae* (VIP = 5.727, *p = 0.02*) and *Actinobacillus* (VIP = 5.483, *p = 0.03*). A further ROC analysis model showed that unclassified *Pasteurellaceae*, unclassified *Neisseriaceae* and *Actinobacillus* had optimized diagnostic and classification capability (AUC = 0.999, *p = 0.021*), followed by *Neisseria* and *Corynebacterium* (AUC = 0.875, *p = 0.083*, **Figure 6D**). Therefore, unclassified *Pasteurellaceae*, unclassified *Neisseriaceae* and *Actinobacillus* were considered to be the most valuable potential biomarkers.

### Predictive Function Gene Analysis

**Figure 7:**
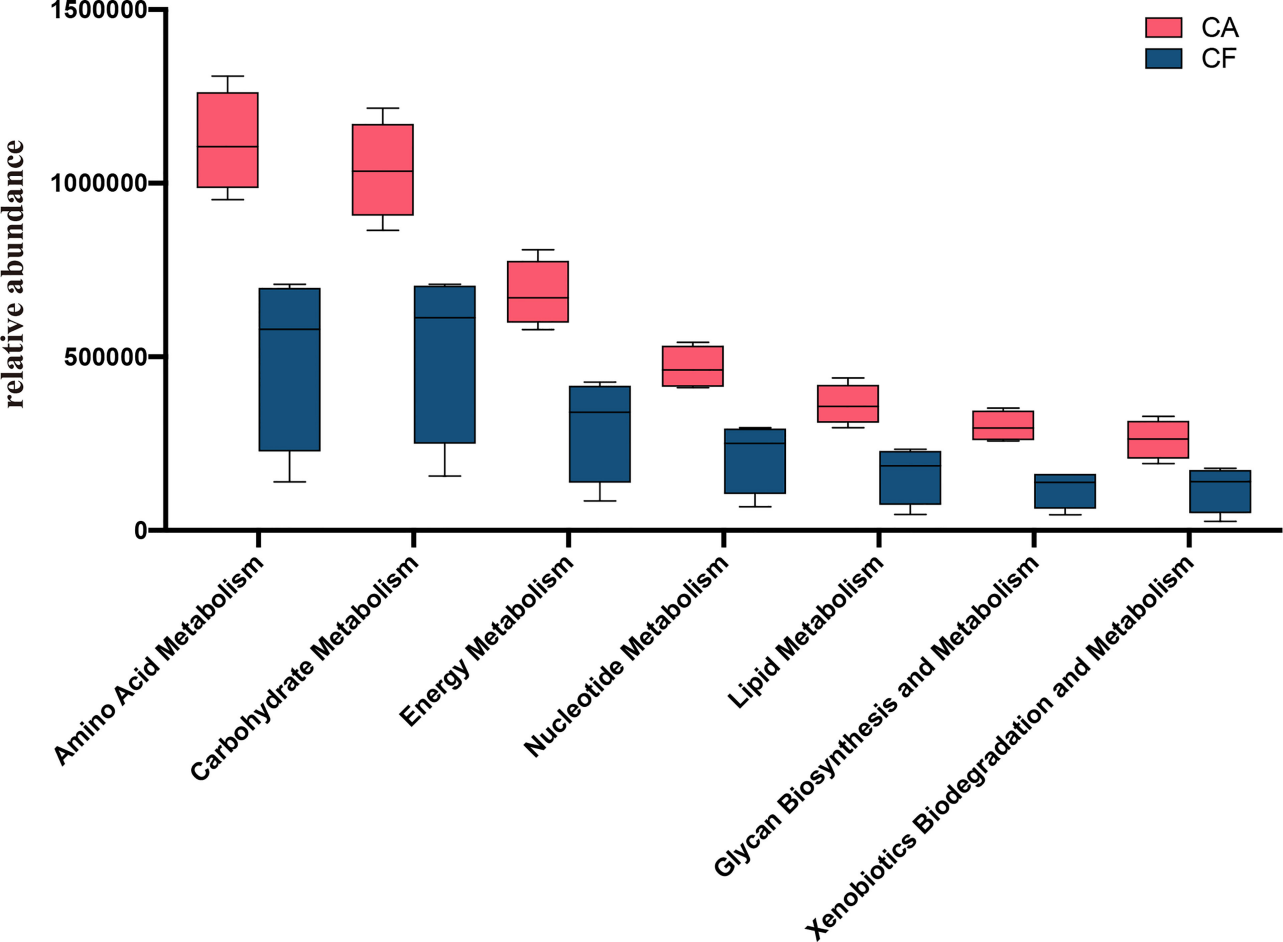
Mann-Whitney U test result of metabolism pathway difference (KEGG level 2). The red box represent CA group and blue box represent CF group. The value of p obtained by Mann-Whitney U test, all the metabolism pathways are significantly different between the two groups (all the *p* = *0.03*).

The functional genes of the samples were analyzed using the Kyoto Encyclopedia of Genes and Genomes (KEGG) database. All the levels of energy related metabolic pathways on KEGG level 2, for example: amino acid metabolism, carbohydrate metabolism, energy metabolism, glycan biosynthesis and metabolism, lipid metabolism, nucleotide metabolism, xenobiotics biodegradation and metabolism in the CA group were significantly higher than those in the CF group (all the *p value = 0.03*, **Figure 7**). We also investigated bacterial functions associated with human diseases which were significantly enriched in the CA group, such as renal cell carcinoma, pathways in cancer, tuberculosis, African trypanosomiasis, chagas disease (American trypanosomiasis), Epithelial cell signaling in Helicobacter pylori infection and Amyotrophic lateral sclerosis (ALS) (**Table S2**).

### Discussion

A comprehensive and thorough investigation of the bacterial diversity of plaque microbiota is essential for understanding their etiologies and for developing effective prevention and treatment strategies of dental caries. The introduction of high-throughput 16S rRNA sequencing has provided new insights into the compositions and structures of microbial communities (Yang et al., 2012). At present, giant panda oral microbes still need to be explored, in order to better classify and identify microorganisms, we have selected the more reported V3 and V4 variable regions which yield the highest accuracies of phylogenetic assignment and Illumina mature system in our study (Xu et al., 2018; Lee et al., 2021).

Different from human plaque research, collection of plaque samples from animals (especially wild animals) is particularly difficult due to their food intake and behavior. In order to get accurate and effective plaque bacterial structure information without using anesthesia, there needs to be a mutual trust relationship between the keepers and giant panda cubs. The use of anesthesia was abandoned for two reasons: 1) sampling after anesthesia may have negative health effects for the animal and reduce trust between animals and keepers, making subsequent research sampling more difficult; 2) the composition of respiratory anesthesia may affect the bacterial structure of oral plaque (Sturgeon et al., 2013). For these reasons, without anesthesia, we obtained 350,420 high-quality sequences with an average of 43,802 sequences per sample, which was much higher than reported in previous human studies and animal studies (Keijser et al., 2008; Ling et al., 2010; Sturgeon et al., 2014). According to Hamady and Knight’s (2009) research, a depth of coverage of approximately 1000 sequences per sample was sufficient to enable detection of species at 1% relative abundance with reasonable accuracy. Thus, the sequencing depth in our study was reasonable and large enough to enable detection of the vast majority of bacterial species in plaque samples. However, there are still some bacteria in the results that cannot be accurately classified on genus level, speculating that aspects of the bacterial community of giant pandas and other wild animals is still unknown.

It is worth mentioning that in our study the bacterial diversity of plaque in giant panda cubs were significantly increased in the group with dental caries (Shannon: CA VS. CF, *p = 0.03*; Simpson: CA VS. CF, *p = 0.03*). In most of the previous studies of dental caries, the microbial diversity of individuals with caries is lower than that of healthy individuals, and the diversity index decreases with severity of dental caries, which supports the notion of the “ecological catastrophe” (Li et al., 2005; Marsh, 2006; Preza et al., 2008). In some reports, however, we found similar results to our study, and therefore we presume that due to the altered oral environment, the relative abundance of beneficial bacteria that support oral health is reduced and core microflora function is disturbed, leading to the colonization of many opportunistic bacteria (Luo et al., 2012). This implies that changes of oral bacterial community structure may be related to the shift from healthy to diseased.

Microbiota can reach an equilibrium by competing for niche and nutrition and mediate colonization resistance against pathogenic bacteria (Chassaing and Cascales, 2018). A suitable relative abundance of different plaque microorganisms contributes to the health of the oral environment, once established, the resident oral microbiota is fairly stable and resilient to exogenous encounters (Marsh and Zaura, 2017). This stability can, however, be lost when environmental stress factors overrule resilience which will cause a colonization of rare bacteria. Our study with PCoA analysis showed that the caries-active samples were more dispersed compare with the caries-free samples, which can be interpreted as an imbalance of the bacterial community.

The core microbiome is considered to be a key component of the basic functions of all organisms, and is enriched, selected and inherited through evolutionary processes (Lundberg et al., 2012; Lemanceau et al., 2017). The OTUs shared between the eight giant panda cubs were determined in order to establish the core microbiome, which was common among the eight samples. In our study, 337 OTUs corresponding to 189 unique genus-level identifications were present in all samples. Here we only reported the 10 bacteria on genus level whose proportion exceeds 1% of all sequences, representing the most core microbiome. The CA group has 45 more unique OTUs than the CI group, which is in agreement with the findings of Schloss et al. (2011), that some extremely low relative abundance of rare species could be detected and might be related to dental caries. In the future, we will increase the sample size and usage of samples from twin cubs and further strengthen the research on core microorganisms.

At the phylum level, 13 different phyla were identified in the plaque samples of both CA and CF groups. Five phylum had relative abundances higher than 1% (**Figure 2A**), of which the phylum Proteobacteria, Bacteroidetes, Firmicutes, Actinobacteria and Fusobacteria have been previously detected in oral microbiota studies of both humans and animals (Jiang et al., 2013; Willis and Gabaldon, 2020). From the phyla relative abundance, the structure of giant panda cubs’ oral microbiota is more similar to that of cats (Sturgeon et al., 2014) and cattle (Borsanelli et al., 2018), followed by kangaroo infants (Chhour et al., 2010) swine (Kernaghan et al., 2012), and then humans, chimpanzees and bonobos (Li et al., 2013), with the most differences with canines (Sturgeon et al., 2013) and marine mammals (Bik et al., 2016). Bacteroidetes was significantly enriched in the CF group and Fusobacteria was significantly enriched in the CA group. By LEfSe test, Fusobacteria and *Fusobacterium* were siginificantly enriched in caries-active cubs. *Fusobacterium* has been reported as one of the most effective co-aggregators of human dental plaque, and was enriched in the CA group (Kolenbrander et al., 1989). *Fusobacterium* could co-aggregated to early colonizers, and express very high autoinducer 2 activity. Supported by the increased sugar metabolism that resulted in increased Glycan biosynthesis in the CA group, may have formed a strong matrix of biofilm which helped bridging bacteria, such as Fusobacteria and Corneybacerium, link the early and late colonizers in the biofilm, ultimately causing caries and caries recurrence (Kalpana et al., 2020).

At the genus level, compared with the CF group, the CA group mainly changed from the replacement of the dominant bacteria. The relative abundance of unclassified *Neisseriaceae* and *Actinobacillus* significantly decreased in the CA group even though they still had a high percentage. The remaining spaces were occupied by *Lautropia*, *Neisseria*, unclassified *Pasteurellaceae*, *Moraxella* and *Canocytophaga*. In the present study, using an LEfSe test, unclassified *Pasteurellaceae*, *Fusobacterium*, *Paenibacillus* and *Leptotrichia* were siginificantly enriched in giant panda cubs with dental caries. Species in the family Pasteurellaceae exhibit urease activity, can survive in an acidic environment and influence the development of dental caries (Morou-Bermudez et al., 2015; Nomura et al., 2020). *Paenibacillus* is used for the prevention of dental caries because of its effect of hydrolyzing the biofilm formed by *Streptococcus mutans* (Kolahi and Abrishami, 2013). We also found cases of oral disease caused by *Paenibacillus* (Al Qarni et al., 2017). We speculate that the enrichment of *Paenibacillus* in the CA group is due to the increased concentration of sugar substrates resulting from the increased intake of carbohydrate components in the diet. *Leptotrichia* has been proved to have high saccharolytic potential, is able to ferment a large variety of mono- and disaccharides to lactic acid, and is well adapted to thrive under conditions that are conducive to the formation of caries (Thompson and Pikis, 2012). Certain *Leptotrichia* species were shown to be negatively associated with elevated urease activity in plaques, which is correlated with dental health, and therefore positively associated with caries (Morou-Bermudez et al., 2015; Richards et al., 2017). According the results of PLS-DA and ROC model analysis, *unclassified Pasteurellaceae*, *unclassified Neisseriaceae* and *Actinobacillus* were considered as a potential biomarker for the diagnosis of dental caries in giant panda cubs. Next step, we will increase the number of samples and further study the potential biomarkers. Besides, dental caries are not caused by a specific pathogenic bacterium, and the intrinsic association between the different dental plaque microorganisms and caries in giant panda cubs needs further in-depth study.

In this study, zoonotic pathogens were identified in each oral sample, particularly Streptococcus. These data suggest that those zoonotic pathogens may be a common commensal in giant pandas. These findings highlight the zoonotic potential of the oral flora of healthy giant pandas and the inherent risks of exposure to saliva and oral exposure.

In this study we compared the differences in metabolic pathways between the CA group and the CF group at KEGG level 3, in addition to deliberately comparing the metabolic pathways between the two groups at KEGG level 2. Not surprisingly, all the metabolism pathways at KEGG level 2 were enriched in the CA group including the top three with the highest relative abundance of amino acid metabolism, carbohydrate metabolism and energy metabolism. As with most previously reported findings, enhanced metabolic pathways are positively associated with the development of caries, especially carbohydrate metabolism (Moye et al., 2014; Strużycka, 2014; Wang et al., 2019).

As the first research of giant panda dental plaque microflora with caries, this study explored the difference of plaque microbiota composition and function of giant panda cubs with and without dental caries, in which the characteristics and core bacteria of those were analyzed. In addition, the differences of dental plaque microflora could be used as a potential biomarker for the diagnosis of dental caries in giant panda cubs. These findings can further deepen the understanding of the panda cubs’ oral cavity microenvironment and provide valuable information for disease treatment and captive management.

## Data Availability Statement

The datasets presented in this study can be found in online repositories. The names of the repository/repositories and accession number(s) can be found in the article/**Supplementary Material**.

## Ethics Statement

The animal study was reviewed and approved by Institutional Animal Care and Use Committee of the Chengdu Research Base of Giant Panda Breeding.

## Author Contributions

RM, RH, J-LG, D-WQ, and Q-GY contributed to the conception of the study. RM, J-LG, and X-YZ performed the experiment. RM, J-LG, D-WQ, and Q-GY contributed significantly to analysis and manuscript preparation. RM, D-WQ, and Q-GY performed the data analyses and wrote the manuscript. S-JC, X-BH, RW, Y-PW, QZ, S-YD, J-CL, and YB helped perform the analysis with constructive discussions. All authors contributed to the article and approved the submitted version.

## Funding

Funding was provided and supported by the National Natural Science Foundation of China (U21A20193, No.31772484); Project of Sichuan Department of Science and Technology (No. 2017JQ0026) and the Chengdu Giant Panda Breeding Research Foundation (No. CPF2017-20).

## Conflict of Interest

The authors declare that the research was conducted in the absence of any commercial or financial relationships that could be construed as a potential conflict of interest.

## Publisher’s Note

All claims expressed in this article are solely those of the authors and do not necessarily represent those of their affiliated organizations, or those of the publisher, the editors and the reviewers. Any product that may be evaluated in this article, or claim that may be made by its manufacturer, is not guaranteed or endorsed by the publisher.

## References

[B1] Al Qarni A. M. M. Joseph M. R. Moadi Y. M. Almaliki A. Y. Hamid M. E. (2017). Localized Severe Gingivitis Caused by Paenibacillus Apiarius in a 28-Year-Old Male Patient: A First Case Report. Int. J. Med. Dental Case Rep. 4 (1), 1–3. doi: 10.15713/ins.ijmdcr.68

[B2] Bik E. M. Costello E. K. Switzer A. D. Callahan B. J. Holmes S. P. Wells R. S.. (2016). Marine Mammals Harbor Unique Microbiotas Shaped by and Yet Distinct From the Sea. Nat. Commun. 7, 10516. doi: 10.1038/ncomms10516 26839246 PMC4742810

[B3] Borsanelli A. C. Lappin D. F. Viora L. Bennett D. Dutra I. S. Brandt B. W.. (2018). Microbiomes Associated With Bovine Periodontitis and Oral Health. Vet. Microbiol. 218, 1–6. doi: 10.1016/j.vetmic.2018.03.016 29685214

[B4] Chassaing B. Cascales E. (2018). Antibacterial Weapons: Targeted Destruction in the Microbiota. Trends Microbiol. 26 (4), 329–338. doi: 10.1016/j.tim.2018.01.006 29452951

[B5] Chhour K. L. Hinds L. A. Jacques N. A. Deane E. M. (2010). An Observational Study of the Microbiome of the Maternal Pouch and Saliva of the Tammar Wallaby, Macropus Eugenii, and of the Gastrointestinal Tract of the Pouch Young. Microbiology (Reading) 156 (Pt 3), 798–808. doi: 10.1099/mic.0.031997-0 19833775

[B6] Cuozzo F. P. Sauther M. L. (2006). Severe Wear and Tooth Loss in Wild Ring-Tailed Lemurs (Lemur Catta): A Function of Feeding Ecology, Dental Structure, and Individual Life History. J. Hum. Evol. 51 (5), 490–505. doi: 10.1016/j.jhevol.2006.07.001 16962643

[B7] Fleming J. L. (1965). The Interplay of Diet and Microbes in the Production of Dental Caries. Dent. Stud. 44 (2), 105–109. doi: 10.1007/978-94-009-3341-5_27 5214871

[B8] Guo M. Chen J. Li Q. Fu Y. Fan G. Ma J.. (2018). Dynamics of Gut Microbiome in Giant Panda Cubs Reveal Transitional Microbes and Pathways in Early Life. Front. Microbiol. 9. doi: 10.3389/fmicb.2018.03138

[B9] Head D. A. Marsh P. D. Devine D. A. (2014). Non-Lethal Control of the Cariogenic Potential of an Agent-Based Model for Dental Plaque. PloS One 9 (8), e105012. doi: 10.1371/journal.pone.0105012 25144538 PMC4140729

[B10] Hull V. Shortridge A. Liu B. Bearer S. Zhou X. Huang J.. (2011). The Impact of Giant Panda Foraging on Bamboo Dynamics in an Isolated Environment. Plant Ecol. 212, 43–54. doi: 10.1007/s11258-010-9800-3

[B11] Jiang W. Zhang J. Chen H. (2013). Pyrosequencing Analysis of Oral Microbiota in Children With Severe Early Childhood Dental Caries. Curr. Microbiol. 67 (5), 537–542. doi: 10.1007/s00284-013-0393-7 23743597

[B12] Jin Y. Chen S. Chao Y. Pu T. Xu H. Liu X.. (2015). Dental Abnormalities of Eight Wild Qinling Giant Pandas (Ailuropoda Melanoleuca Qinlingensis), Shaanxi Province, China. J. Wildl. Dis. 51 (4), 849–859. doi: 10.7589/2014-12-289 26280879

[B13] Jin Y. Lin W. Huang S. Zhang C. Pu T. Ma W.. (2012). Dental Abnormalities in Eight Captive Giant Pandas (Ailuropoda Melanoleuca) in China. J. Comp. Pathol. 146 (4), 357–364. doi: 10.1016/j.jcpa.2011.08.001 21906751

[B14] Kalpana B. Prabhu P. Bhat A. H. Senthilkumar A. Arun R. P. Asokan S.. (2020). Bacterial Diversity and Functional Analysis of Severe Early Childhood Caries and Recurrence in India. Sci. Rep. 10 (1), 1–15. doi: 10.1038/s41598-020-78057-z 31913322 PMC6959339

[B15] Keijser B. J. Zaura E. Huse S. M. van der Vossen J. M. Schuren F. H. Montijn R. C.. (2008). Pyrosequencing Analysis of the Oral Microflora of Healthy Adults. J. Dent. Res. 87 (11), 1016–1020. doi: 10.1177/154405910808701104 18946007

[B16] Kernaghan S. Bujold A. R. MacInnes J. I. (2012). The Microbiome of the Soft Palate of Swine. Anim. Health Res. Rev. 13 (1), 110–120. doi: 10.1017/S1466252312000102 22853946

[B17] Kolahi J. Abrishami M. (2013). Mutanase-Containing Chewing Gum: A New Potential Approach for Prevention of Dental Caries. Dental Hypotheses 4 (2), 53. doi: 10.4103/2155-8213.113010

[B18] Kolenbrander P. E. Andersen R. N. Moore L. V. (1989). Coaggregation of Fusobacterium Nucleatum, Selenomonas Flueggei, Selenomonas Infelix, Selenomonas Noxia, and Selenomonas Sputigena With Strains From 11 Genera of Oral Bacteria. Infect. Immun. 57 (10), 3194–3203. doi: 10.1128/iai.57.10.3194-3203.1989 2777378 PMC260789

[B19] Lee E. Park S. Um S. Kim S. Lee J. Jang J.. (2021). Microbiome of Saliva and Plaque in Children According to Age and Dental Caries Experience. Diagnostics (Basel) 11 (8), 1324. doi: 10.3390/diagnostics11081324 34441259 PMC8393408

[B20] Lemanceau P. Blouin M. Muller D. Moënne-Loccoz Y. (2017). Let the Core Microbiota be Functional. Trends Plant Sci. 22 (7), 583–595. doi: 10.1016/j.tplants.2017.04.008 28549621

[B21] Li Y. Ku C. Xu J. Saxena D. Caufield P. (2005). Survey of Oral Microbial Diversity Using PCR-Based Denaturing Gradient Gel Electrophoresis. J. Dental Res. 84 (6), 559–564. doi: 10.1177/154405910508400614

[B22] Li J. Nasidze I. Quinque D. Li M. Horz H. P. Andre C.. (2013). The Saliva Microbiome of Pan and Homo. BMC Microbiol. 13, 204. doi: 10.1186/1471-2180-13-204 24025115 PMC3848470

[B23] Ling Z. Kong J. Jia P. Wei C. Wang Y. Pan Z.. (2010). Analysis of Oral Microbiota in Children With Dental Caries by PCR-DGGE and Barcoded Pyrosequencing. Microb. Ecol. 60 (3), 677–690. doi: 10.1007/s00248-010-9712-8 20614117

[B24] Loyola J. P. Leyva P. (1990). [Dental Caries in an Animal Model]. Rev. ADM 47 (4), 190–194. 2257272

[B25] Lundberg D. S. Lebeis S. L. Paredes S. H. Yourstone S. Gehring J. Malfatti S.. (2012). Defining the Core Arabidopsis Thaliana Root Microbiome. Nature 488 (7409), 86–90. doi: 10.1038/nature11237 22859206 PMC4074413

[B26] Luo A. H. Yang D. Q. Xin B. C. Paster B. J. Qin J. (2012). Microbial Profiles in Saliva From Children With and Without Caries in Mixed Dentition. Oral. Dis. 18 (6), 595–601. doi: 10.1111/j.1601-0825.2012.01915.x 22458262

[B27] Malmsten A. Dalin A. M. Pettersson A. (2015). Caries, Periodontal - Disease, Supernumerary Teeth and Other Dental Disorders in Swedish Wild Boar (Sus Scrofa). J. Comp. Pathol. 153 (1), 50–57. doi: 10.1016/j.jcpa.2015.04.003 25979683

[B28] Manville A. M. (1978). Ecto-And Endoparasites of the Black Bear in Northren Wisconsin. J. Wildl. Dis. 14 (1), 97–101, 105. doi: 10.7589/0090-3558-14.1.97 633522

[B29] Marsh P. D. (2006). Dental Diseases–are These Examples of Ecological Catastrophes? Int. J. Dent. Hyg. 4 Suppl 1, 3–10; discussion 50-12. doi: 10.1111/j.1601-5037.2006.00195.x 16965527

[B30] Marsh P. D. Zaura E. (2017). Dental Biofilm: Ecological Interactions in Health and Disease. J. Clin. Periodontol. 44 (S18), S12–S22. doi: 10.1111/jcpe.12679 28266111

[B31] Maruyama N. Maruyama F. Takeuchi Y. Aikawa C. Izumi Y. Nakagawa I. (2014). Intraindividual Variation in Core Microbiota in Peri-Implantitis and Periodontitis. Sci. Rep. 4, 6602. doi: 10.1038/srep06602 25308100 PMC4194447

[B32] Morou-Bermudez E. Rodriguez S. Bello A. S. Dominguez-Bello M. G. (2015). Urease and Dental Plaque Microbial Profiles in Children. PloS One 10 (9), e0139315. doi: 10.1371/journal.pone.0139315 26418220 PMC4587978

[B33] Moye Z. D. Zeng L. Burne R. A. (2014). Fueling the Caries Process: Carbohydrate Metabolism and Gene Regulation by Streptococcus Mutans. J. Oral. Microbiol. 6 (1), 24878. doi: 10.3402/jom.v6.24878

[B34] Munson M. A. Banerjee A. Watson T. F. Wade W. G. (2004). Molecular Analysis of the Microflora Associated With Dental Caries. J. Clin. Microbiol. 42 (7), 3023–3029. doi: 10.1128/JCM.42.7.3023-3029.2004 15243054 PMC446285

[B35] Nomura R. Matayoshi S. Otsugu M. Kitamura T. Teramoto N. Nakano K. (2020). Contribution of Severe Dental Caries Induced by Streptococcus Mutans to the Pathogenicity of Infective Endocarditis. Infect. Immun. 88 (7), e00897-00819. doi: 10.1128/IAI.00897-19 32312765 PMC7309618

[B36] Preza D. Olsen I. Aas J. A. Willumsen T. Grinde B. Paster B. J. (2008). Bacterial Profiles of Root Caries in Elderly Patients. J. Clin. Microbiol. 46 (6), 2015–2021. doi: 10.1128/JCM.02411-07 18385433 PMC2446847

[B37] Richards V. P. Alvarez A. J. Luce A. R. Bedenbaugh M. Mitchell M. L. Burne R. A.. (2017). Microbiomes of Site-Specific Dental Plaques From Children With Different Caries Status. Infect. Immun. 85 (8), e00106-00117. doi: 10.1128/IAI.0120106-17 28507066 PMC5520424

[B38] Schloss PD. Gevers D. Westcott SL. (2011). Reducing the Effects of PCR Amplification and Sequencing Artifacts on 16S rRNA-based Studies. PLoS One 6 (12), e27310. doi: 10.1371/journal.pone.0027310 22194782 PMC3237409

[B39] Selwitz R. Ismail A. Pitts N. (2007). Dental Caries. Lancet 369, 51–59. doi: 10.1016/S0140-6736(07)60031-2 17208642

[B40] Shuangyun L. Yongjin C. Min Z. Xinyu L. (2009). Morphological and Functional of Giant Panda (Ailuropoda Melanoleuca) Dentition From the Qinling Mountains. Acta. Theriol. Sin. 29 (3), 332. doi: 10.1016/S1874-8651(10)60059-2

[B41] Stromquist A. Fahlman A. Arnemo J. M. Pettersson A. (2009). Dental and Periodontal Health in Free-Ranging Swedish Brown Bears (Ursus Arctos). J. Comp. Pathol. 141 (2-3), 170–176. doi: 10.1016/j.jcpa.2009.05.001 19539950

[B42] Strużycka I. (2014). The Oral Microbiome in Dental Caries. Pol. J. Microbiol. 63 (2), 127. doi: 10.33073/pjm-2014-018 25115106

[B43] Sturgeon A. Pinder S. L. Costa M. Weese J. S. (2014). Characterization of the Oral Microbiota of Healthy Cats Using Next-Generation Sequencing. Vet. J. 201 (2), 233-239. doi: 10.1016/j.tvjl.2014.01.024

[B44] Sturgeon A. Stull J. Costa M. Weese J. (2012). Metagenomic Analysis of the Canine Oral Cavity as Revealed by High-Throughput Pyrosequencing of the 16S rRNA Gene. Vet. Microbiol. 162. (2-4), 891–898. doi: 10.1016/j.vetmic.2012.11.018 23228621

[B45] Sturgeon A. Stull J. W. Costa M. C. Weese J. S. (2013). Metagenomic Analysis of the Canine Oral Cavity as Revealed by High-Throughput Pyrosequencing of the 16S rRNA Gene. Vet. Microbiol. 162 (2-4), 891–898. doi: 10.1016/j.vetmic.2012.11.018 23228621

[B46] Tanzer J. M. Livingston J. Thompson A. M. (2001). The Microbiology of Primary Dental Caries in Humans. J. Dent. Educ. 65 (10), 1028–1037. doi: 10.1002/j.0022-0337.2001.65.10.tb03446.x 11699974

[B47] Teng F. Yang F. Huang S. Bo C. Xu Z. Z. Amir A.. (2015). Prediction of Early Childhood Caries *via* Spatial-Temporal Variations of Oral Microbiota. Cell Host Microbe 18 (3), 296–306. doi: 10.1016/j.chom.2015.08.005 26355216

[B48] Thompson J. Pikis A. (2012). Metabolism of Sugars by Genetically Diverse Species of Oral Leptotrichia. Mol. Oral. Microbiol. 27 (1), 34–44. doi: 10.1111/j.2041-1014.2011.00627.x 22230464 PMC3257818

[B49] Verdon T. J. Mitchell R. J. van Oorschot R. A. (2014). Swabs as DNA Collection Devices for Sampling Different Biological Materials From Different Substrates. J. Forensic Sci. 59 (4), 1080–1089. doi: 10.1111/1556-4029.12427 24502761

[B50] Wang J. (1961). Dental Caries of Fossil Ailuropoda of Kwangsi. Vert. Palas. 4, 330–339.

[B51] Wang A. H. Li M. Li C. Q. Kou G. J. Zuo X. L. Li Y. Q. (2016). Human Colorectal Mucosal Microbiota Correlates With its Host Niche Physiology Revealed by Endomicroscopy. Sci. Rep. 6, 21952. doi: 10.1038/srep21952 26916597 PMC4768150

[B52] Wang Y. Wang S. Wu C. Chen X. Duan Z. Xu Q.. (2019). Oral Microbiome Alterations Associated With Early Childhood Caries Highlight the Importance of Carbohydrate Metabolic Activities. MSystems 4 (6), e00450–00419. doi: 10.1128/mSystems.00450-19 31690590 PMC6832018

[B53] Wenker C. J. Stich H. Müller M. Lussi A. (1999). A Retrospective Study of Dental Conditions of Captive Brown Bears (Ursus Arctos Spp.) Compared With Free-Ranging Alaskan Grizzlies (Ursus Arctos Horribilis). J. Zoo Wildl. Med. 30 (2), 208–221. 10484135

[B54] Willis J. R. Gabaldon T. (2020). The Human Oral Microbiome in Health and Disease: From Sequences to Ecosystems. Microorganisms 8 (2), 308. doi: 10.3390/microorganisms8020308

[B55] Xie G. Chain P. Lo C. C. Liu K. L. Gans J. Merritt J.. (2010). Community and Gene Composition of a Human Dental Plaque Microbiota Obtained by Metagenomic Sequencing. Mol. Oral. Microbiol. 25 (6), 391–405. doi: 10.1111/j.2041-1014.2010.00587.x 21040513 PMC2975940

[B56] Xuanzhen L. Mingxi L. Jianqiu Y. Zhihe Z. Xiangming H. Jingchao L.. (2005). Composition of Captive Giant Panda Milk. Zoo Biol. 24 (4), 393–398. Published in affiliation with the American Zoo and Aquarium Association. doi: 10.1002/zoo.20056

[B57] Xu L. Chen X. Wang Y. Jiang W. Wang S. Ling Z.. (2018). Dynamic Alterations in Salivary Microbiota Related to Dental Caries and Age in Preschool Children With Deciduous Dentition: A 2-Year Follow-Up Study. Front. Physiol. 9. doi: 10.3389/fphys.2018.00342

[B58] Yang F. Zeng X. Ning K. Liu K. L. Lo C. C. Wang W.. (2012). Saliva Microbiomes Distinguish Caries-Active From Healthy Human Populations. ISME J. 6 (1), 1–10. doi: 10.1038/ismej.2011.71 21716312 PMC3246229

[B59] Yin X. Gu X. Yin T. Wen H. Gao X. Zheng X. (2016). Study of Enteropathogenic Bacteria in Children With Acute Diarrhoea Aged From 7 to 10 Years in Xuzhou, China. Microb. Pathog. 91, 41–45. doi: 10.1016/j.micpath.2015.11.027 26657723

